# Optimising remote monitoring for cardiac implantable electronic devices: a UK Delphi consensus

**DOI:** 10.1136/heartjnl-2024-324167

**Published:** 2024-09-18

**Authors:** Shumaila Ahmad, Sam Straw, John Gierula, Eleri Roberts, Jason Collinson, Matthew Swift, Chris Monkhouse, Lucy Broadhurst, Annabel Allan, Haqeel A Jamil, Anne Dixon, Paula Black, Ian Pinnell, Hannah Law, Natalie Archer, Fozia Ahmed, Maria F Paton

**Affiliations:** 1 Leeds Institute for Cardiovascular and Metabolic Medicine, University of Leeds, Leeds, UK; 2 Manchester University NHS Foundation Trust, Manchester, UK; 3 Barts Health NHS Trust, London, UK; 4 Great Western Hospital Foundation NHS Trust, Swindon, UK; 5 Rotherham NHS Foundation Trust, Rotherham, UK; 6 Harrogate and District NHS Foundation Trust, Harrogate, North Yorkshire, UK; 7 Airedale NHS Foundation Trust, Keighley, UK; 8 Leeds Teaching Hospitals NHS Trust, Leeds, UK; 9 Blackpool Teaching Hospitals NHS Foundation Trust, Blackpool, UK; 10 Oxford University Hospitals NHS Foundation Trust, Oxford, UK; 11 NHS Lothian, Edinburgh, UK; 12 Southern Health and Social Care Trust, Portadown, Armagh, UK; 13 Manchester Heart Centre, Manchester University NHS Foundation Trust, Manchester, UK

**Keywords:** Telemedicine, Electrophysiology, Quality of Health Care

## Abstract

**Background:**

Remote monitoring (RM) is recommended for the ongoing management of patients with cardiac implantable electronic devices (CIEDs). Despite its benefits, RM adoption has increased the workload for cardiac rhythm management teams. This study used a modified Delphi method to develop a consensus on optimal RM management for adult patients with a CIED in the UK.

**Methods:**

A national steering committee comprising cardiac physiologists, cardiologists, specialist nurses, support professionals and a patient representative developed 114 statements on best RM practices, covering capacity, support, service delivery, coordination and clinical escalation. An online questionnaire was used to gather input from UK specialists, with consensus defined as ≥75% agreement.

**Results:**

Between 16 October 2023 and 4 December 2023, 115 responses were received. Of the statements, 79 (69%) achieved high agreement (≥90%), 20 (18%) showed moderate agreement (75%–89%) and 15 (13%) did not achieve consensus. The highest agreement focused on patient education and support, while the lowest concerned workload distribution.

**Conclusions:**

There is strong agreement on best practices for RM of CIEDs among UK healthcare professionals. Key recommendations include ensuring patient access, providing adequate resources, adopting new working methods, enhancing patient education, establishing clear clinical escalation pathways and standardising national policies. Implementing these best practices, tailored to local capabilities, is essential for effective and equitable RM services across the UK.

**WHAT IS ALREADY KNOWN ON THIS TOPIC**

- Remote monitoring (RM) for those with cardiac implantable electronic devices (CIEDs) should now be a key component of cardiac rhythm management (CRM) services; however, it poses a number of challenges for clinical teams, including a potential increased work burden. The rapid development of RM services that have been established has resulted in great variation and unequal access for those with CIEDs across the UK.

**WHAT THIS STUDY ADDS**

- This study demonstrates the significant growth in knowledge and expertise with regard to RM among many CRM services. There is a high level of agreement in what constitutes best practices for RM services in the UK, with recommendations focused on delivering access for every patient, adequate service resources, the adoption of new ways of working, improved patient education and support, clear pathways for clinical escalation and enhanced national standardisation of policies.

**HOW THIS STUDY MIGHT AFFECT RESEARCH, PRACTICE OR POLICY**

- This expert consensus provides practical recommendations for clinical teams and services to strive for when developing and delivering RM services for patients with CIEDs, specifically within the UK. Not only will it affect daily practice and guide resources and access, but it will also serve to highlight evidence gaps where further investigation is required.

## Introduction

 Cardiac implantable electronic devices (CIEDs), including pacemakers, implantable cardioverter-defibrillators (ICDs), cardiac resynchronisation therapy and implantable loop recorders, are routinely used in the management of cardiac arrhythmia and chronic heart failure.^1 2^ Until recently, conventional in-person follow-up was required to monitor device functionality and diagnostic findings.^3^ Remote monitoring (RM) technologies have been commercially available for some time, yet their widespread adoption was accelerated during the COVID-19 pandemic and has led to a paradigm shift in CIED follow-up^4^ with significant implications for patients, carers and the National Health Service (NHS).

Data transmitted by RM can be scheduled, alert based or patient initiated.^4^ RM has the potential advantages of enhanced patient convenience, early escalation of adverse diagnostic findings, possible reductions in healthcare costs due to fewer in-person visits and improved patient adherence to follow-up schedules.^5^ However, with the widespread adoption of RM, there has been anecdotally an increase in workload for healthcare professionals, due to the requirement for specialist trained staff to review and process complex data which would not have otherwise been available. In addition, only 6.6% of scheduled transmissions are reported to result in any change to patient management, yet each download takes 5–30 min to review, report and action.^6^ RM might therefore result in inefficiencies in service delivery at a time where there are significant challenges to staff recruitment and retention.^7^ Consequently, many departments are unable to offer RM services for their entire patient cohort, or at all, resulting in healthcare inequalities.

To support the successful development and delivery of services in the UK, we sought to collate an expert consensus on best practices for CIED RM among multidisciplinary healthcare professionals specialising in cardiac rhythm management (CRM).

## Methods

This study used a modified Delphi methodology^8^ to establish a consensus of expert opinions by delivering sequential, anonymously completed, structured questionnaires between 16 October and 4 December 2023.

### Delphi steering committee

Committee composition was designed to be inclusive of all relevant professional backgrounds and practice environments across the UK. Individuals were sought who had experience of providing or being aligned with an RM service for CIEDs for a minimum of 2 years, or involvement in health policymaking or research where RM was the focus, or were a patient interested in or had lived experience of CIED therapy and could commit to participate to project completion. Potential participants were identified from publicly available information including published work, publicly available websites, reports and policy documents, via social media (eg, X, LinkedIn) or by recommendation from confirmed participants.

16 potentially eligible committee members were invited by MFP by email, of which 15 agreed to participate. The steering committee comprised cardiac physiologists (7), cardiologists (consultant physicians) (2), heart failure and cardiac rhythm nurse specialists (2), CRM support professionals (1), clinical academics (2) and a patient representative (table 1). The committee included eight women and health professionals who practise across England, Scotland and Northern Ireland with a high level of experience.

**Table 1:** Steering committee demographics

Demographics	Panellists, n, (n=15)
Sex	
Male	7 (47%)
Female	8 (53%)
Professional role	
Consultant cardiologist	2 (13%)
Clinical academic	2 (13%)
Clinical cardiac scientist/physiologist	7 (47%)
Cardiac specialist nurse	2 (13%)
CRM support worker	1 (7%)
Patient representative	1 (7%)
Practice region	
South West of England	1 (7%)
South of England	1 (7%)
Greater London	2 (13%)
North of England	6 (40%)
North West England	2 (13%)
Scotland	2 (13%)
Northern Ireland	1 (7%)
Years of clinical practice	
0–10	1 (7%)
11–15	6 (40%)
16–20	4 (27%)
>20	3 (20%)

CRMcardiac rhythm management

### Patient and public involvement

Patients and the public have been involved in every stage. The study design and dissemination plan were developed with the Leeds cardiovascular research facility patient and public involvement and engagement group. One member was an integral and equal member of the steering committee, contributing to all discussions, survey design, result evaluation and manuscript review, sharing their lived experience of RM.

### Delphi survey

**Figure 1:**
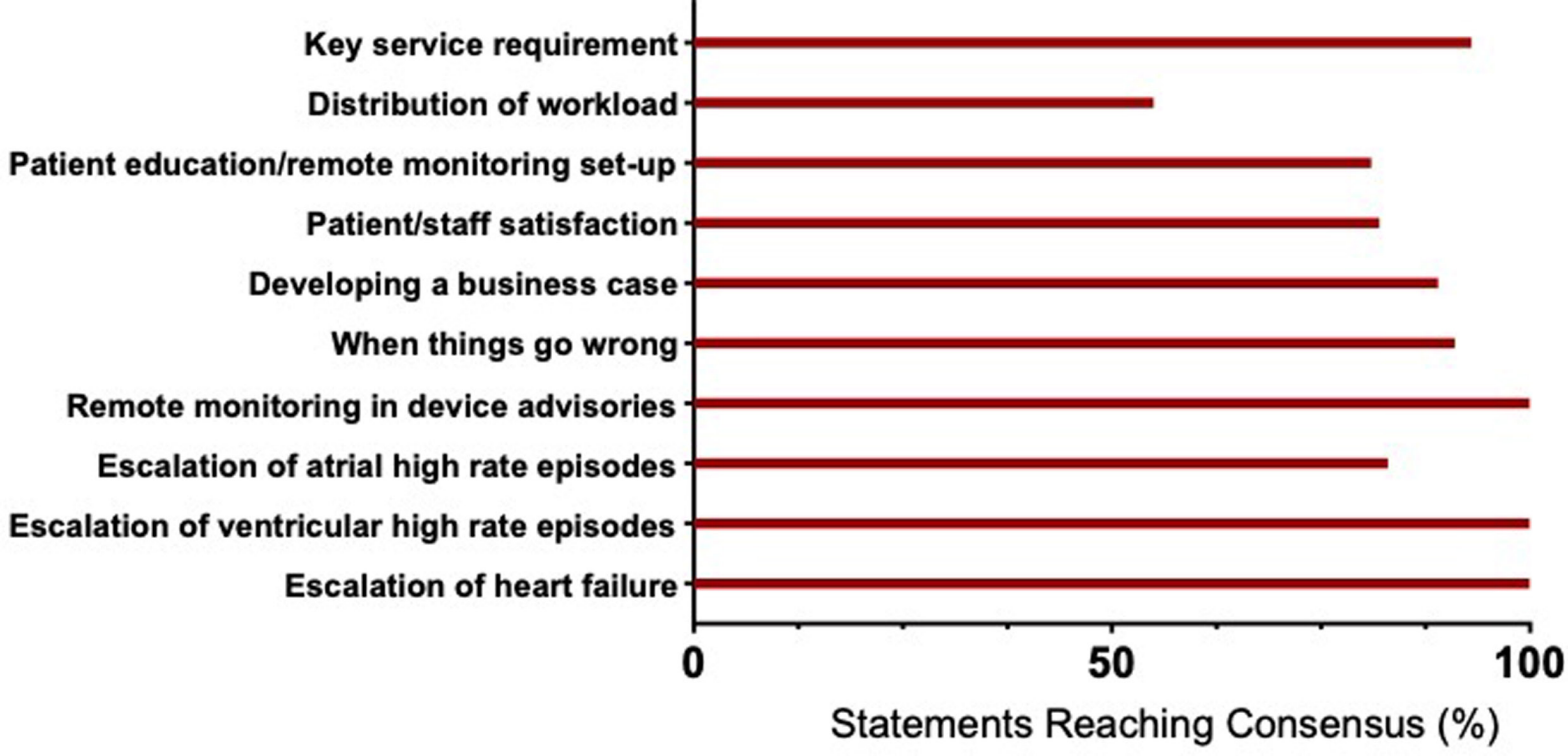
Consensus across the key themes of remote monitoring. Summary figure of the percentage of statements reaching consensus per key theme of the survey on remote monitoring.

Pre-reading was provided to steering committee participants, providing a summary of published evidence on RM in CIED, prior to an online survey development meeting. During this meeting, three topics were discussed; challenges in RM, what is working well and exploring opportunities for improvement. Themes were highlighted from by the study team to capture essential aspects of best practice and finalised by the steering committee; service requirements (A), distribution of workload (B), set-up and education (C), user satisfaction (D), developing a business case (E), what to do when things go wrong (F), RM in the context of device advisories (G), the assessment of atrial high rates (H), ventricular high rates (I) and heart failure (J) (figure 1). The patient representative advocated framing the Delphi study on the assumption that RM may offer an acceptable and convenient alternative to in-person visits.

A total of 114 statements were developed across these 10 key themes (online supplemental table 1), proposed by the steering committee during the survey development meeting. All steering committee members contributed and reviewed statements. The statements were designed into an online survey with capacity for respondents to ‘agree’ or ‘disagree’ on a binary scale for each statement. Respondents were asked to deliberate the statements in reference to what they would consider to be approaches to delivering successful RM services within their clinical departments and within a real-world scenario. The anonymous online survey was disseminated via the British Heart Rhythm Society to their members and by the British Heart Foundation newsletter between 16 October 2023 and 4 December 2023. The committee adopted a pragmatic approach and considered all invited participants as experts given their role in delivering care to patients with CIEDs. Consensus was decided a priori as ≥75% agreement and was further defined as demonstrating ‘high’ (≥90%) and ‘moderate’ (75%–89%) agreement.

Anonymous survey findings were collated and reviewed by the steering committee during a second consensus meeting. Due to the levels of agreement, it was decided that further consensus rounds were not required. Recommendations for best practice were developed by the steering committee after review of the survey results and were reviewed by all members prior to publication.

## Results

### Delphi survey

**Figure 2:**
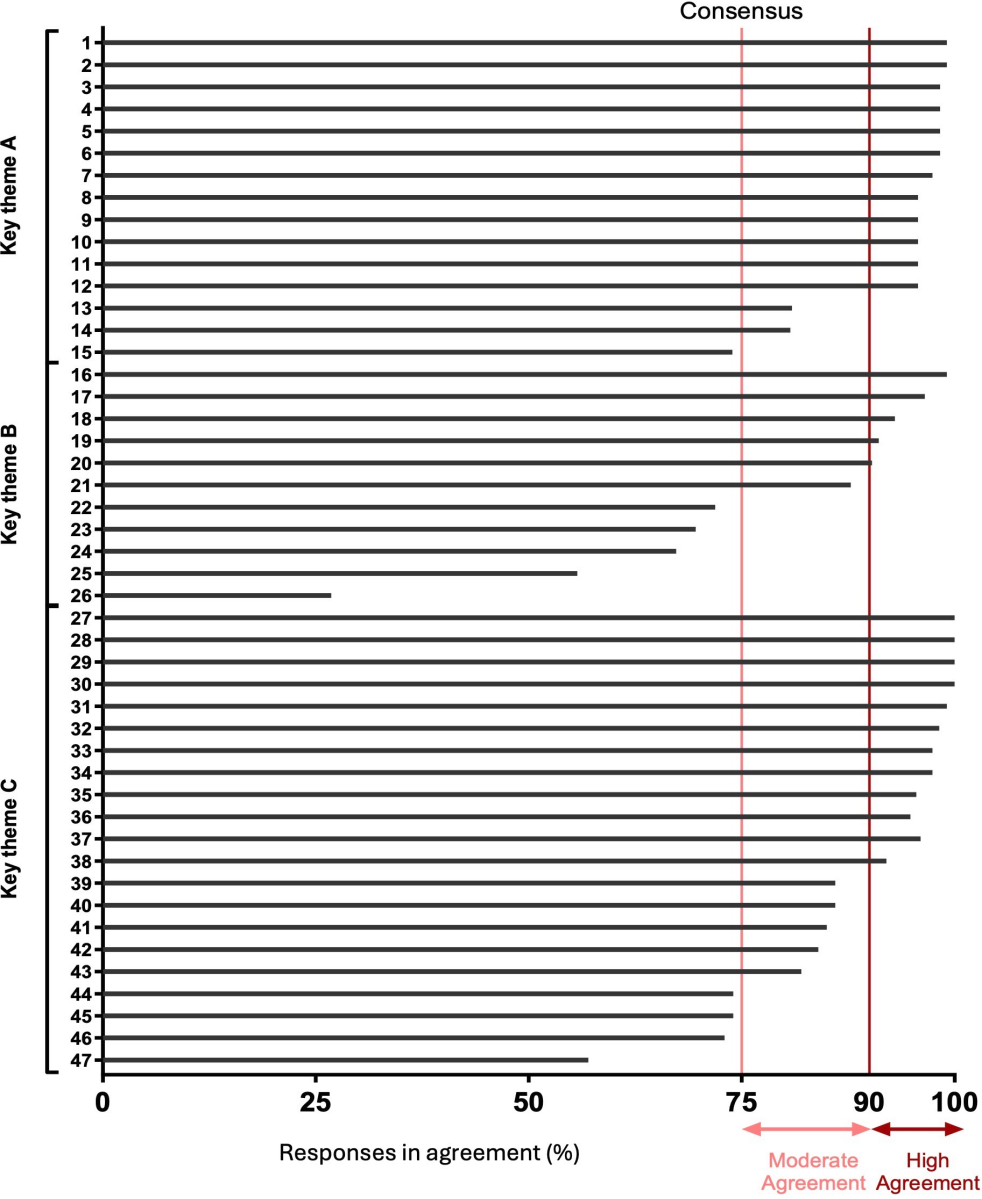
Consensus according to each survey statement for key themes A–C. The level of agreement for each survey statement for themes: key service requirements, distribution of workload, patient education/remote monitoring set-up.

A total of 115 individuals responded, taking a median time of 15 (IQR 12–24) minutes to complete the survey, with a total of 12 997 out of 13 110 (99%) potential statement responses achieved. Of the statements, 99 (87%) reached the consensus threshold of ≥75%, with 79 (69%) achieving high (≥90%) consensus and 20 (18%) achieving moderate (75%–89%) consensus (figures2^4^). Only 15 (13%) statements did not achieve consensus, pertaining to multiple themes (A–F, H).

**Figure 3:**
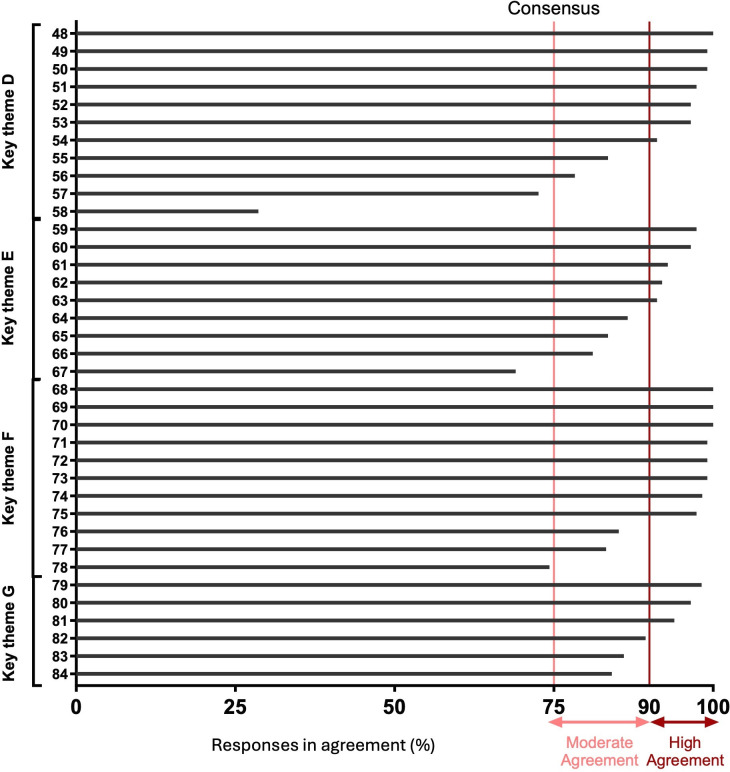
Consensus according to each survey statement for key themes D–G. The level of agreement for each survey statement for themes: patient/staff satisfaction, developing a business case, when things go wrong, remote monitoring in device advisories.

**Figure 4:**
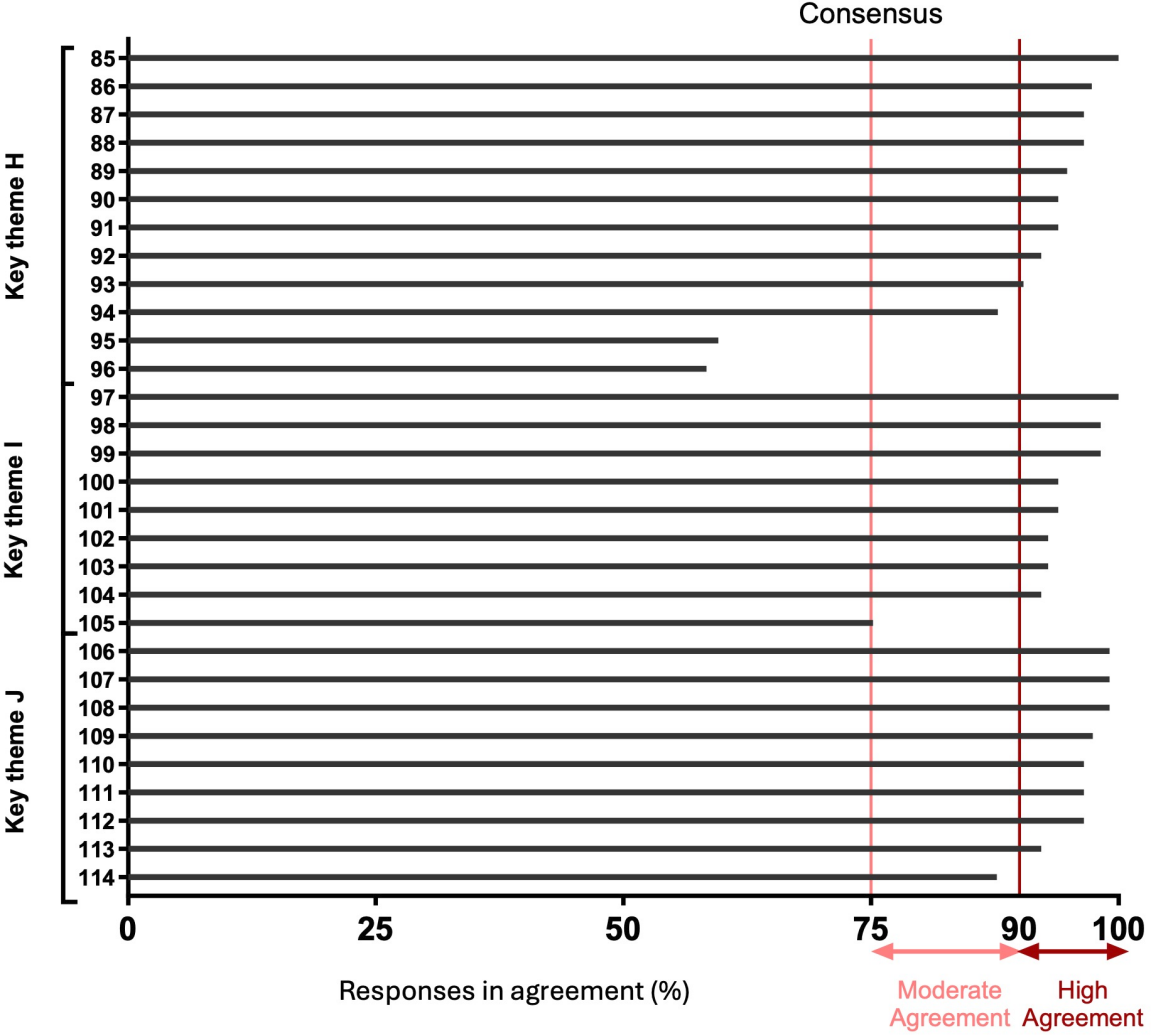
Consensus according to each survey statement for key themes H–J. The level of agreement for each survey statement for themes: escalation of atrial high rate episodes, escalation of ventricular high rate episodes, escalation of heart failure.

### Statements with the highest consensus

Interested stakeholders should review and consider the study findings in their entirety; however, we highlight 10 (9%) statements which achieved unanimous consensus.

Four of the top ranked statements focus on patient education and support for connectivity:

1. Patients should be provided with contact details to assist with connectivity.
2. Manufacturers should provide education resources to patients about their specific RM device.
3. Manufacturers should provide education resources that are easy to understand.
4. Patients should have easy support from knowledgeable staff.

Multiple highest ranked statements define best practice in adverse events:

1. Each department should have standard operation procedures to determine action and escalation required for RM alerts.
2. Standard operating procedures should specify when patients should be referred to a specialist arrhythmia pathway.
3. All healthcare professionals involved in RM should have access to the appropriate medical records.
4. RM services should have capacity to urgently review patients in clinic as required.
5. Driver and Vehicle Licensing Agency recommendations following ICD therapy should be communicated to the patient.
6. RM should be offered to those patients who find it difficult to attend in-person clinics.

Of note, statements in the themes of RM in the context of advisories (G), assessment of ventricular high rates (I) and assessment of heart failure (J), achieved consensus for all statements.

### Statements which did not reach consensus

Relatively few areas of disagreement were observed. The lowest agreement was observed for regional centres to manage RM (27% agreement). The theme with the least consensus (55%) described the distribution of workload (B).

Several unrelated cases gained 74% agreement (statements 15, 44, 45, 78) and were therefore not deemed to have reached consensus (online supplemental table 1).

### Best practice recommendations

The steering committee interpreted the Delphi results within the context of their own experience and synthesised best practice recommendations. Agreement levels per statement are available in online supplemental table 1.

### There should be equitable access to RM across the UK

RM has become an integral component of the management of people with CIEDs to ensure optimal device functionality and detect adverse diagnostic findings. Patients and professionals agree that best practice requires services to be accessible to all patients across the UK, delivered 7 days per week, with review and management of alert-based findings within 24 hours. Furthermore, RM should be distributed and connected at implant, with connectivity ensured within 4 weeks.

### RM services must have adequate resources

Services should be supported by appropriate infrastructure. An integrated and automated digital system, permitting timely transfer of summarised data with access to electronic health records is regarded as best practice. There should be dual screen workstations, some of the costs of which can likely be offset by waste reduction. Using remote transmissions for patients who have not attended their in-clinic appointments minimises duplicate appointments. In addition, all RM device costs should be incorporated in CIED costs upfront, ensuring equitable access. Patient and staff satisfaction are also important considerations and should be audited regularly.

### New ways of working to meet the challenges of delivering RM services

Dedicated and protected clinical and administrative RM provision should be achieved, aligned with industry support to ensure effective workload distribution. While there are no restrictions on professional requirements of those reviewing RM data, the consensus view was that there must be oversight by a CRM accredited healthcare professional. RM permits flexible working and an opportunity for autonomy, positively effecting morale. This should be balanced within a broader workload including in-clinic follow-up to maintain of skills. Currently, three dedicated full-time equivalent staff per 1000 RM patients is accepted as sufficient to meet workforce requirements, based on recommendations from an international expert consensus statement and calculator.^4 9^ Interestingly, consensus was not reached for a time allocation of 20 min for scheduled transmission review, however it is not clear whether this was seen as too little, or too much time.

Knowledgeable support staff should take a leading role in delivering patient and carer education to ensure appropriate and consistent information. Device manufacturers should take responsibility for developing and maintaining user-friendly educational resources, confirming device connectivity and providing helpline support, all of which need to be accessible and inclusive, for example, to meet the requirements of people with sight or hearing needs, and those necessitating language options.

There was a lack of consensus with regard to using third party companies to triage transmissions, and strong disagreement with the utilisation of regional departments.

### Patients should be empowered to get the most from their device

No consensus on the process of consent was reached, although there was consensus that information should be given prior to implantation. Follow-up processes should be explained clearly, and allow for patient follow-up and contact preference. A non-compliance protocol should be implemented: the first episode of non-compliance should lead to seeking patient contact, while a second episode should lead to an in-clinic appointment if appropriate.

If a device incapable of automated RM is in situ, regular scheduled follow-ups should be adopted instead, and regardless of follow-up mode, patients should receive annual communication from their CRM team.

### A pathway should exist for escalating clinical findings

RM services should have contribution from the wider multidisciplinary team (including heart failure and arrhythmia specialists) to deliver optimised care. There should be clear, evidence-based, standard operating protocols to determine action and escalation for alert-based transmissions. Alerts should have maximum programmability across manufacturers to ensure individualised optimisation and to future proof for translation of new evidence-based practices.

Should alert-based diagnostics reach the threshold for action, there should be in-clinic acute capacity and a clear process for obtaining clinical and symptomatic review of the patient within guideline-directed timescales. Staff reviewing the patient should be trained in advanced communication and have referral pathways for psychological services.

### Standardised national processes should exist in the management of device advisories

In the advent of a CIED advisory, health professionals are calling for standardised national approaches. There is high consensus that manufacturers should be readily available for support and be able to highlight potential patients affected. There was also consensus in favour of reimbursement should follow-up intensity be increased by manufacturer guidance, and that manufacturers should support connectivity.

## Discussion

### Results in context

Although there is a growing body of literature advocating RM for patients with CIEDs, limited guidance exists to direct service structure and protocols. Our study is the first to obtain consensus on best practice in RM of CIEDs within the UK. The UK has a unique healthcare environment, with the NHS providing healthcare free at the point of access; however, it is a cost-constrained healthcare environment with its own unique set of challenges. As such, consensus documents from other healthcare systems may be difficult to apply. Our consensus document was led by a multidisciplinary expert steering committee, representing services from urban and rural settings, as well as secondary and tertiary care. We identified areas of expert agreement and those which require further research. Despite a lack of empirical evidence in many aspects of service delivery, consensus was reached in the majority of statements, allowing us to propose several key recommendations.

### Key findings

First, every centre caring for patients with CIEDs should have an RM service and should strive for a 7-day service. Second, there should be a range of healthcare professionals integrated within the service using evidence-based protocols for escalation of adverse findings. Information on RM should be provided to the patient prior to implant where possible, and patients should be supported to specify their follow-up preferences. Finally, manufacturer support is vital and particularly welcome in ensuring accessible educational materials, ongoing connectivity and in the advent of a device advisory.

Our study findings echo some opinions outlined in the European Heart Rhythm Association, Heart rhythm Association, Asia Pacific Heart Rhythm Society and Latin American Heart rhythm Society consensus on RM,^4^ yet is distinct given its design, its committee diversity and specific focus on UK practice. Where possible, our study emphasises practical recommendations, supported by respondents’ considerations that these should be implementable in real-world contemporary clinical practice, although some recommendations are likely to be considered aspirational. It is important to acknowledge service development challenges which may be causing disparity in services and access. Notably, there was a lack of strong agreement in specific staffing requirements and clinical escalation protocols.

While the consensus identified services should be delivering 7-day care, agreement was only moderate, likely indicating the perceived difficulty in achieving these targets. There is some scope to unify regional teams, potentially within integrated care systems to distribute workload evenly, share expertise and achieve provision for full-time RM services. Nevertheless, collaborative regional working was not a concept supported within the consensus, suggesting further investigations are needed to develop optimal working practices.

Divisive areas of practice were also observed within the clinical scenario themes, specifically in the best practice for anticoagulation pathways following atrial high rate episodes. This likely represents the emerging and seemingly conflicting evidence base; two recently published clinical trials reached divergent results.^10 11^ Further comment on thresholds for escalation following alert-based detection of potentially clinically significant findings were outside the scope of this study, although committee discussions highlighted a need for further research, and a concern that device data may be underleveraged. This was supported by the consensus opinion that device data should be available for diagnostic assessment and that alert criteria should be programmable to permit translation of new evidence to practice.

Some statements attempted to specifically address challenging areas, such as time requirements for transmission review, for which statements on both estimated allocation and whether there was sufficient evidence to guide allocation failed to reach consensus, inevitably impacting service provision. Another is the use of industry partners, where there seemed to be unease around utilisation compared with international consensus.^4^ However, clearly RM services are reliant on both clinical teams and device manufacturers working synergistically for the benefit of our patients. Importantly though, the steering committee unanimously agreed establishing RM provision for all appropriate patients is a priority. Failure to adopt RM services risks the diversion of resources to patients in higher need or at higher risk of adverse clinical outcomes.

### Strengths and limitations

This consensus used systematic consensus-building methodology to determine best practice in RM of those with CIEDs. Statements were developed by an expert multidisciplinary professional and patient steering committee, including representation across UK nations, to incorporate experts from varying health and funding models. The consensus survey was the largest undertaken to date and demonstrated a high agreement rate among statements, adding weight to the validity of recommendations developed. Responses were sought from across the UK, and the survey was available in open access to gather a wide range of opinions. All survey responses were entirely anonymous, and no demographic information was collected; therefore, it is not possible to conclude whether our sample was truly representative of all those involved with RM in the UK.

Pilot testing of the survey was not undertaken, and we assumed that all potential respondents would have expertise in this area. However, to mitigate against the risk of misunderstanding, open text comments were invited during the survey which were carefully reviewed by the steering committee, none of which suggested misinterpretation of any survey question. This study undertook one round of open consensus due to the high rate of agreement, yet no responses warranting adaption of the statements were received.

## Conclusions

This consensus on best practice for the RM of CIEDs is based on expert opinion from a multidisciplinary steering committee, validated by healthcare professionals specialised in CRM. The level of agreement was generally high, which might be unexpected given the current discrepancy in service provision. Guidance for clinical teams to adopt the recommended best practice protocols, aligned to local capabilities, is a key first step in ensuring effective and equitable CIED RM services across the UK.

## supplementary material

10.1136/heartjnl-2024-324167 online supplemental file 1

## Data Availability

Data are available on reasonable request. All data relevant to the study are included in the article or uploaded as online supplemental information.
