# Supplementary material for: Optimising remote monitoring for cardiac implantable electronic devices: a UK Delphi consensus
Source: Heart. 2024 Sep 18;110(23):e324167. doi: 10.1136/heartjnl-2024-324167 (PMC11671946; doi:10.1136/heartjnl-2024-324167)
Supplement: online supplemental file 1 [file heartjnl-110-23-s001.pdf]

Supplementary Table 1: Consensus statements and level of agreement		
**Key Service Requirement**		Level of Agreement
1	Time for reviewing and reporting remote monitoring data should be protected.	99
2	Remote monitoring services should have relationships and access to specialist clinical teams across cardiology.	99
3	There should be alert management in place to protect remote monitoring clinic time.	98
4	Patients should be aware of the remote services cover provided by their centre.	98
5	Remote monitoring data should be reported appropriately to document both scheduled and unscheduled workload.	98
6	All people with the following devices should have access to a remote monitoring service.<br>• Loop Recorder<br>• Pacemaker<br>• CRT<br>• ICD	98
7	Audit should be an integral part of the remote monitoring service to understand local service requirements.	97
8	Remote monitoring reporting should be overseen by an accredited healthcare professional.	96
9	Remote monitoring services should have dedicated clinical support.	96
10	Remote monitoring services should have dedicated administrative support.	96
11	All cardiac device follow-up centres should offer a remote monitoring service.	96
12	Remote monitoring reporting should be overseen by an accredited healthcare professional.	96
13	Remote monitoring alerts should be managed within 24 hours	81
14	Remote monitoring should be a 7-day service.	81
15	A consultant level healthcare professional should be responsible for overall care of patients with remote follow-up capabilities.	74
**Distribution of Workload**		
16	There should be support services from industry to ensure patient connectivity.	99
17	There should be clear guidelines for clinical and non-clinical roles within remote monitoring services	97

18	Patient queries relating to remote monitoring should be initially triaged by service support staff.	93
19	Remote monitoring services should have strong local links across the Integrated Care System.	91
20	Loop recorder data can be monitored by staff trained in ambulatory ECG monitoring.	90
21	Remote monitoring consent and set-up should be performed by service support staff.	88
22	Remote follow-up clinics should allocate 20 minutes to review and report each transmission.	72
23	An accredited healthcare professional should supervise the assessment of remote data from patients with loop recorder devices.	70
24	There is evidence to suggest time allocation to review and report each transmission.	67
25	Third party companies (including manufacturers) could be involved in the triage of unscheduled remote monitoring data.	56
26	There should be regional centres to manage remote monitoring.	27
**Patient education/remote monitoring set-up**		
27	Patients should be provided with contact details to assist with connectivity.	100
28	Manufacturers should provide education resources to patients about their specific remote monitoring device.	100
29	Manufacturers should provide education resources that are easy to understand.	100
30	Patients should have easy support from knowledgeable staff.	100
31	Patients should be provided with relevant information about what they should do in an emergency.	99
32	Patients should be informed that remote monitoring is not an emergency management system.	98
33	Remote monitoring device education should be provided in multiple media formats to ensure accessibility which should be available via the manufacturer.	97
34	Patients should have access to their manufacturer's remote monitoring helpline.	97
35	Carers/family should be involved in patient education on remote monitoring devices and services.	96
36	Connectivity should be ensured within 4 weeks once a patient has received their remote monitoring device.	95
37	Patients should be advised to have their device ID with them at all times	96

38	There should be national standardized information to provide to patients prior to consent for remote monitoring.	92
39	Patients should be provided with remote monitoring information before the implant.	86
40	Patients should be offered remote monitoring on the day of their device implant.	86
41	Patients should be assessed for suitability according to local protocols prior to being offered remote monitoring.	85
42	Patients should be advised not take remote monitors abroad.	84
43	Patient education should be led by remote monitoring service support staff.	82
44	Patient written consent for remote monitoring should be taken and stored in patient records.	74
45	Patients should be given a choice for the type of monitor for remote services.	74
46	Manufacturers should provide refresher education to all patients with remote monitoring.	73
47	Patients should be encouraged to have an app based remote monitor.	57
**Patient/Staff Satisfaction**		
48	Remote monitoring should be offered to those patients who find it difficult to attend in-person clinics.	100
49	Remote monitoring training should be provided to all staff involved in remote monitoring clinics.	99
50	Peer support should be readily available for staff within remote monitoring services.	99
51	Remote monitoring should aid flexible working for clinical staff which can help with staff recruitment and retention.	97
52	Cardiac rhythm management specialists should have a balanced workload undertaking both remote monitoring services and in-clinic follow ups.	97
53	Remote monitoring allows flexibility of workplace, i.e. various working sites and environments.	97
54	Patient satisfaction with remote monitoring services should be regularly audited.	91
55	Patients should have contact with cardiac rhythm management services every 12 months regarding their cardiac device regardless of their follow-up pathway.	84
56	Patients should be given the opportunity to choose their preferred type of follow up and contact process (telephone, letter, face to face)	78

57	Verbal contact should be made between remote services and patients every 24 months	73
58	Staff morale is negatively affected by remote monitoring.	29
**Developing a Business Case**		
59	Remote monitoring device costs should be included in all costs for the cardiac devices.	97
60	An integrated and automated IT system should be considered to aid accurate and efficient data collection and transfer to the patient electronic health record.	97
61	Remote monitoring workstations should have dual computer screens.	93
62	Remote monitoring allows more equal access cardiac device follow-up for patients.	92
63	There should be a Display Screen Equipment assessment for staff working onsite or from home undertaking remote monitoring services.	91
64	Remote monitoring reduces wasted clinic time for multiple appointments due to non-attendance by substituting for a remote download.	87
65	There should be involvement of the wider cardiac multidisciplinary team in remote monitoring services.	84
66	There should be 3 dedicated full time equivalent remote monitoring service members of staff per 1000 patients on remote monitoring (following HRS/EHRA/APHRS/LAHRS guidance [4])	81
67	Remote monitoring reduces the costs of resources required for face-to-face clinics.	69
**What to do when things go wrong?**		
68	Each department should have standard operation procedures to determine action and escalation required for remote monitoring alerts.	100
69	All healthcare professionals involved with remote monitoring should have access to the appropriate medical records.	100
70	Remote monitoring services should have capacity to urgently review patients in clinic as required.	100
71	There should be a pathway in place for dealing with advisory notifications.	99
72	Scheduled in-clinic follow-ups should be in place for patients who are not compliant or not able to use remote monitoring.	99
73	Patient should be aware of the process of how to raise a complaint with regards to remote monitoring.	99

74	There should be a standard operating procedure following identification of a potential deceased patient on remote monitoring.	98
75	There should be a local process in place for non-compliance.	97
76	All remote monitoring transmissions should be documented appropriately within the patient health record.	85
77	Standard procedure for noncompliance should be as follows:<br>o 1x failed download: patient to be contacted,<br>o 2x failed download: patient to rebook face to face.	83
78	Patients should be able to send unscheduled alerts in case of emergency.	74
**Remote monitoring in context of device advisories**		
79	Manufacturer support should be readily available for escalation.	98
80	Manufacturer remote monitoring portals should highlight patients with device advisories.	97
81	There should be a standardised national approach to the management of patients with device advisories.	94
82	If device follow-up is intensified on the recommendation of the manufacturer, it should be reimbursed.	89
83	Remote monitoring NHS Trust population data should be easily accessible via the manufacturer portal.	86
84	If there is a device advisory, the manufacturer should be responsible for connectivity management of the patients effected.	84
**Escalation of atrial high rate episodes**		
85	Standard operating procedures should specify when patients should be referred to a specialist arrhythmia pathway.	100
86	National and international guidance for AF management should include a statement on cardiac device remote monitoring services.	97
87	AF alerts should be programmable and personalisable across all manufacturers to the same degree.	97
88	Duration and burden of AF at which referral for anticoagulation is recommended should be based on evidence from randomised controlled trials.	97
89	There should be a clear standard operating procedure for programming AF alerts.	95
90	All patients with detected AF should have risk factor optimisation for stroke avoidance.	94
91	The presence of AF should be reported to primary care physicians to allow for accurate clinical assessment of each patient.	94
92	All programming of alerts should be evidence based.	92

93	AF alerts should be programmed off if the information obtained would not change clinical management.	90
94	Patients with AF alerts should have their symptoms assessed.	88
95	The decision to anticoagulate is the responsibility of primary care physicians.	60
96	The decision to anticoagulate is the responsibility of specialist healthcare professionals.	58
**Escalation of ventricular high rate episodes**		
97	DVLA recommendations following therapy should be communicated to the patients.	100
98	Standard operating procedures should specify when patients should be referred to a specialist arrhythmia pathway.	98
99	Patients who have received an ICD shock should have access psychological support.	98
100	The detection of sustained ventricular arrhythmia (both above and below ICD detection) should prompt a clinical review.	94
101	Patients with devices incapable of automatic alerts should have regular scheduled follow-ups.	94
102	For non-sustained ventricular tachycardia, patients should have a clinical review if symptomatic.	93
103	Alert criteria for non-sustained ventricular tachycardia are restrictive and should be more programmable across manufacturers.	93
104	Physiologists reviewing these patients should have training in advanced communication to support patients.	92
105	Patients who have received a single shock and are not compromised should not have to attend face-to-face clinic.	75
**Escalation of heart failure**		
106	Patients with diagnosed heart failure should be reviewed as per national guidance.	99
107	There should be a clear standard operating procedure to assess patients with heart failure alerts.	99
108	There should be clinical training provided to clinicians who review heart failure alerts.	99
109	There should be a standard operating procedure for managing low biventricular pacing alerts.	97
110	A heart failure multidisciplinary team should be integrated in remote monitoring of cardiac device patients.	97

111	Heart failure diagnostic alerts should be personalized to each patient.	97
112	There should be a standard operating procedure for programming alerts off and this should be clearly documented within the patient's health record.	97
113	Patients with more than one heart failure alert criteria met should have their symptom status reviewed and be considered for clinical review.	92
114	Remote monitoring should be considered for patients receiving end-of-life care.	88

CRT; cardiac resynchronization therapy, ICD; internal cardioverter defibrillator, ECG; electrocardiogram, HRS; Heart Rhythm Society, EHRA; European Heart rhythm Society, APHRS; Asia Pacific Heart Rhythm Society, LAHRS; Latin American Heart Rhythm Society, AF; atrial fibrillation
